# Development and External Validation of a Preoperative Nomogram for Predicting Lateral Pelvic Lymph Node Metastasis in Patients With Advanced Lower Rectal Cancer

**DOI:** 10.3389/fonc.2022.930942

**Published:** 2022-07-08

**Authors:** Lei Zhang, Feiyu Shi, Chenhao Hu, Zhe Zhang, Junguang Liu, Ruihan Liu, Junjun She, Jianqiang Tang

**Affiliations:** ^1^ Department of General Surgery, The First Affiliated Hospital, Xi’an Jiaotong University, Xi’an, China; ^2^ Center for Gut Microbiome Research, Med-X Institute, The First Affiliated Hospital of Xi’an Jiaotong University, Xi’an, China; ^3^ Department of General Surgery, Peking University First Hospital, Beijing, China; ^4^ Department of Colorectal Surgery, National Cancer Center/National Clinical Research Center for Cancer/Cancer Hospital, Chinese Academy of Medical Sciences and Peking Union Medical College, Beijing, China

**Keywords:** nomogram, advanced lower rectal cancer, lateral pelvic lymph node, lateral pelvic lymph node metastasis, magnetic resonance imaging

## Abstract

**Background:**

The preoperative prediction of lateral pelvic lymph node (LPLN) metastasis is crucial in determining further treatment strategies for advanced lower rectal cancer patients. In this study, we established a nomogram model to preoperatively predict LPLN metastasis and then externally validated the accuracy of this model.

**Methods:**

A total of 287 rectal cancer patients who underwent LPLN dissection were included in this study. Among them, 200 patients from the Peking University First Hospital were included in the development set, and 87 patients from the First Affiliated Hospital of Xi’an Jiaotong University were included in the independent external validation set. Multivariate logistic regression analysis was used to develop the nomogram. The performance of the nomogram was assessed based on its calibration, discrimination, and clinical utility.

**Results:**

Five factors (differentiation grade, extramural vascular invasion, distance of the tumor from the anal verge, perirectal lymph node status, and largest short-axis diameter of LPLN) were identified and included in the nomogram. The nomogram developed based on the analysis showed robust discrimination with an area under the receiver operating characteristic curve (AUC) of 0.878 (95% CI, 0.824–0.932). The validation set showed good discrimination with an AUC of 0.863 (95% CI, 0.779–0.948). Decision curve analysis showed that the nomogram was clinically useful.

**Conclusions:**

The present study proposed a clinical-imaging nomogram with a combination of clinicopathological risk factors and imaging features. After external verification, the predictive power of the nomogram model was satisfactory, and it is expected to be a convenient, visual, and personalized clinical tool for assessing the risk of LPLN metastasis in advanced lower rectal cancer patients.

## Introduction

Lateral pelvic lymph node (LPLN) metastasis has been reported as the major cause of local recurrence after curative resection in patients with advanced lower rectal cancer (ALRC), given that approximately 50% of the local recurrences occur in the lateral pelvic sidewall area (1, 2). It is well known that the management of LPLN in patients with ALRC is controversial and nonstandardized, with significant geographical differences. However, with advances in surgical techniques and the improved understanding of LPLN, some consensus views have been gradually reached. On the one hand, neoadjuvant chemoradiation therapy (nCRT) with total mesorectal excision (TME) alone is insufficient to prevent local recurrence for patients with ALRC, and the addition of LPLN dissection (LPLND) results in a significantly lower local recurrence rate (3, 4). On the other hand, routine LPNLD for all ALRC patients would be inappropriate, and selectively performing LPNLD in specific subgroups of patients would bring practical clinical benefits (5, 6). Thus, identifying the patient subgroup that would truly benefit from LPLND is clinically important to determine the therapeutic strategy prior to treatment.

Several studies have reported some clinicopathological factors that predict LPLN metastasis before surgery (7, 8). Although these risk factors presented a significant association with pathological LPLN metastasis, they lacked sensitivity or specificity and were unable to quantify the risk in the preoperative assessment of LPLN metastasis. At present, an increasing number of studies are using imaging features based on magnetic resonance imaging (MRI) or computed tomography (CT), such as lymph node size, spiculated or indistinct borders, and mottled heterogeneous patterns, to preoperatively evaluate the status of LPLN (8–10). Nevertheless, the diagnostic efficacy of these studies was inconsistent. They have not been applied in the clinical setting for the selection of patients for whom LPLND can be omitted. Hence, we need to further develop more powerful and sensitive diagnostic tools to improve the diagnostic accuracy in predicting LPLN metastasis. Combining imaging features and clinicopathological risk factors for predicting LPLN metastasis may be a more effective diagnostic approach.

Nomograms are considered reliable graphical calculating models that can accurately quantify and predict individual risk events by combining all known independent risk factors (11). Nomograms have been widely established to assist in developing personalized treatment and follow-up management strategies for several cancers, such as small cell lung cancer (12), soft-tissue sarcomas (13), and prostate cancer (14).

Hence, in this study, we sought to develop a comprehensive nomogram incorporating MRI imaging features and clinicopathologicak factors to help quantify the individual risk of LPLN metastasis in patients with ALRC. Additionally, we assessed the predictive accuracy and clinical utility of the nomogram and validated it in an external cohort.

## Methods

### Patient Selection

**Figure 1:**
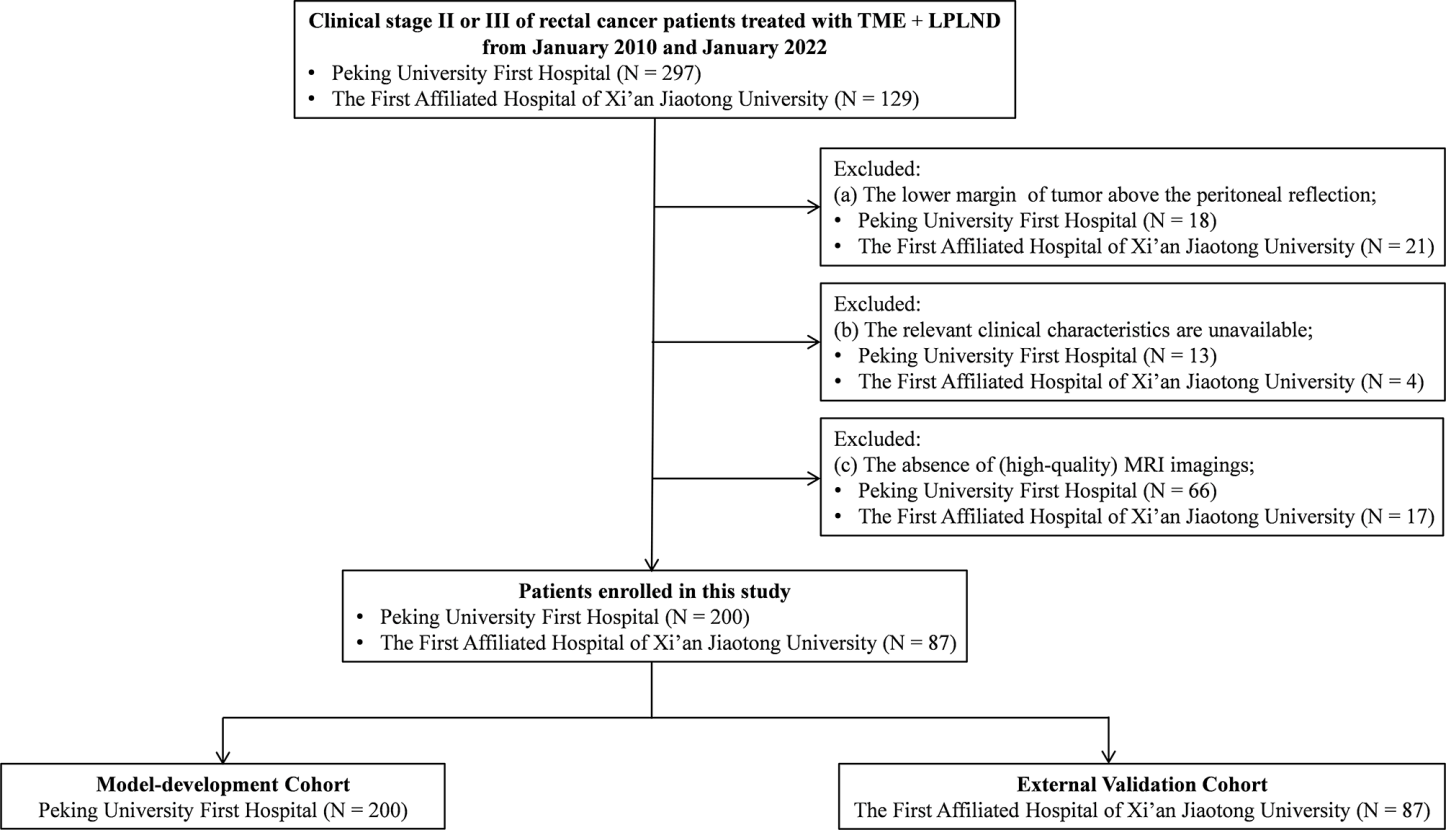
Flow diagram. LPLND, lateral pelvic lymph node dissection; TME, total mesorectal excision; MRI, magnetic resonance imaging.

This retrospective multicenter study was performed at two public tertiary medical centers in China (Peking University First Hospital and the First Affiliated Hospital of Xi’an Jiaotong University). All consecutive patients who underwent TME+LPLND for rectal cancer were screened between January 2010 and January 2022. The inclusion criteria were patients who (a) underwent TME and LPLND for histologically confirmed primary rectal cancer and (b) had a clinical stage of stage II or III. The exclusion criteria included patients (a) with the distal margin of the tumor above the peritoneal reflection; (b) lacking other relevant clinicopathological data in their medical records; and (c) without preoperative MRI. A total of 200 eligible patients at Peking University First Hospital were enrolled and allocated to the model development cohort. Within the same time period, a total of 87 patients who underwent TME+ LPLND at the First Affiliated Hospital of Xi’an Jiaotong University were enrolled as the external validation cohort (**Figure 1**). The protocol of this retrospective study was approved by the Ethics and Human Subject Committee of Peking University First Hospital and the First Affiliated Hospital of Xi’an Jiaotong University (2019-ZD-04).

### Clinicopathological and Imaging Characteristic Assessment

To identify variables associated with LPLN metastasis, the following preoperative clinicopathological characteristics were reviewed and collected from both hospitals: age, sex, body mass index (BMI), grade of tumor differentiation, gross classification of tumor, extramural vascular invasion (EMVI), tumor size, distance from anal verge, cT stage, perirectal lymph node status, short-axis diameter of the largest LPLN, preoperative carcinoembryonic antigen (CEA) level, and preoperative carbohydrate antigen 19-9 (CA19-9) level. The gross classification and differentiation grade of the tumor were assessed by colonoscopy and tissue biopsy. The preoperative imaging features of the tumor were assessed by MRI. All patients had undergone 1.5 or 3.0 T pelvic MRI with pelvic phased-array coils before surgery. If the patient underwent nCRT, the MRI assessment was based on preneoadjuvant MRI. The images were taken in the sagittal, coronal, and axial planes. The cranial border of the field of view was L5, and the caudal border was below the anal canal. The diameter of the lymph node was measured on T2-weighted axial MRI scans of the pelvis, acquired in 4- to 5-mm slices.

The MRIs of all included patients were re-evaluated by two experienced gastrointestinal radiologists. They were asked to make the diagnosis of T stage (T1/T2/T3/T4), tumor size, perirectal lymph node status (positive/negative), the distance of the tumor from the anal verge, and EMVI (positive/negative) for each patient, and a consensus for each disagreed case was determined after discussion. Perirectal lymph nodes with a short-axis diameter of ≥5 mm were defined as positive. Moreover, we measured the short-axis diameter of the largest lymph node located in the lateral pelvic region. The optimal cutoff value of the short-axis diameter in the present study was set at 7 mm, which was determined by the maximum Youden index of the receiver operating characteristic (ROC) curve and is shown in the **Supplementary Methods**. The images were zoomed at the MRI workstation, and measurements were made with workstation electronic calipers.

### Treatment Strategy and Pathology

The treatment strategies for the patients with ALRC were similar in both centers. The standard surgical approach includes open or minimal TME+LPLND. Some of the patients were discussed in a multidisciplinary treatment meeting to decide on the application of nCRT. Unilateral or bilateral LPLND depends on the preoperative imaging findings in the lateral pelvic region. The procedure involved the complete removal of the lateral pelvic lymph nodes and fatty tissues, including the internal iliac nodes, obturator nodes, common iliac nodes, and/or external iliac nodes, with preservation of the bilateral hypogastric nerve and the pelvic nerve plexus. All lymph nodes were dissected from the fresh specimen, and their locations according to the classification by the Japanese Society of Cancer of the Colon and Rectum were documented prospectively.

### Statistical Analysis

Age, BMI, and tumor size were considered continuous variables. Sex, grade of differentiation, EMVI, the distance of the tumor from the anal verge, cT stage, perirectal lymph node status, preoperative CEA level, preoperative CA19-9 level, and short-axis diameter of LPLN were considered categorical variables. For comparisons between groups, Student’s *t*-test was used for continuous variables, and the *χ^2^* test was used for categorical variables.

To develop a well-calibrated and convenient nomogram model for predicting the risk of LPLN metastasis, our nomogram was built using the model development cohort with 200 patients and then validated using 87 patients in the external validation group. We performed a univariate analysis in the development set to determine significant covariates of LPLN metastasis. Significant covariates were then used in a backward stepwise multivariate logistic regression model to determine predictors of pathologically positive LPLN. The nomogram was built based on the results of multivariate logistic regression. The model’s performance was assessed using a calibration curve and the area under the ROC curve (AUC). Decision curve analysis (DCA) was used to evaluate the clinical utility of the nomogram based on net benefits at each risk threshold probability in the development and validation datasets. A *p*-value <0.05 was considered statistically significant. Statistical analysis was performed by using SPSS^®^ version 25.0 (IBM, Armonk, New York, USA) and R software version 3.4.0 (R Foundation for Statistical Computing).

## Results

### Clinicopathological Characteristics of the Patients

A total of 287 patients with ALRC were enrolled in this study, 200 of whom comprised the development cohort and 87 of whom comprised the external validation cohort. All clinicopathological characteristics of both cohorts are summarized in **Table 1**. In terms of the preoperative clinicopathological characteristics of the development cohort and validation cohort, except for gross classification (*p* = 0.016), there were no significant differences in age, sex, BMI, grade of differentiation, EMVI, the distance of the tumor from the anal verge, cT stage, perirectal lymph node status, preoperative CEA level, preoperative CA19-9 level, or largest short-axis diameter of LPLN (**Table 1**). There was no statistically significant difference in the rate of LPLN metastases between the two cohorts (28.0% vs. 24.1%, *p* = 0.497).

**Table 1:** The characteristics of patients in model-development and validation cohorts.

Characteristics	Development cohort				Validation cohort			*p*-value
	Total (n=200)	LPLN positive (n=56)	LPLN negative (n=144)		Total (n=87)	LPLN positive (n=21)	LPLN negative (n=66)	
Age (years)	54.9±12.6	54.9±12.6	57.0±12.0		58.7±12.0	59.4±12.1	58.4±12.0	0.151
Sex (*n*)								0.158
Male	128 (64.0)	27 (48.2)	101 (70.1)		48 (55.2)	7 (33.3)	41 (62.1)	
Female	72 (36.0)	29 (51.8)	43 (29.9)		39 (44.8)	14 (66.7)	25 (37.9)	
BMI (kg/m^2^)	23.4±3.4	23.3±3.5	23.5±3.4		23.9±3.6	23.7±3.7	24.7±3.4	0.252
Grade of differentiation (*n*)								0.785
Well/moderate	144 (72.0)	27 (48.2)	117 (81.3)		64 (73.6)	12 (57.1)	52 (78.8)	
Poor/worse	56 (28.0)	29 (51.8)	27 (18.7)		23 (26.4)	9 (42.9)	14 (21.2)	
Gross classification (*n*)								**0.016**
Protuberant type	29 (14.5)	3 (5.4)	26 (18.1)		23 (26.4)	6 (28.6)	17 (25.8)	
Ulcerative type	171 (85.5)	53 (94.6)	118 (81.9)		64 (73.6)	15 (71.4)	49 (74.2)	
EMVI (*n*)								0.508
Negative	121 (60.5)	10 (17.9)	111 (77.1)		49 (56.3)	5 (23.8)	44 (66.7)	
Positive	79 (39.5)	46 (82.1)	33 (22.9)		38 (43.7)	16 (76.2)	22 (33.3)	
The size of tumor (cm)	5.0±2.2	5.1±2.3	5.0±2.2		4.8±1.7	4.7±1.8	4.9±1.4	0.451
Distance from anal verge (*n*)								0.115
<5cm	130 (65.0)	45 (80.4)	85 (59.0)		48 (55.2)	16 (76.2)	32 (48.5)	
≥5cm	70 (35.0)	11 (19.6)	59 (41.0)		39 (44.8)	5 (23.8)	34 (51.5)	
cT stage (*n*)								0.636
T1-2	41 (20.5)	6 (10.7)	35 (24.3)		20 (23.0)	3 (14.3)	17 (25.8)	
T3-4	159 (79.5)	50 (89.3)	109 (75.7)		67 (77.0)	18 (85.7)	49 (74.2)	
Perirectal lymph nodes status (*n*)								0.531
Negative	100 (50.0)	11 (19.6)	89 (61.8)		40 (46.0)	3 (14.3)	37 (56.1)	
Positive	100 (50.0)	45 (80.4)	55 (38.2)		47 (54.0)	18 (85.7)	29 (43.9)	
Preoperative CEA level (*n*)								0.645
<5 ng/ml	123 (61.5)	29 (51.8)	94 (65.3)		56 (64.4)	14 (66.7)	42 (63.6)	
≥5 ng/ml	77 (38.5)	27 (48.2)	50 (34.7)		31 (35.6)	7 (33.3)	24 (36.4)	
Preoperative CA19-9 level (*n*)								0.347
<37 U/ml	153 (76.5)	36 (64.3)	117 (81.3)		62 (71.3)	13 (61.9)	49 (74.2)	
≥37 U/ml	47 (23.5)	20 (35.7)	27 (18.8)		25 (28.7)	8 (38.1)	17 (25.8)	
The largest short-axis diameter of LPLN (*n*)								0.363
<7 mm	133 (66.5)	18 (25.5)	112 (77.8)		53 (60.9)	6 (28.6)	47 (71.2)	
≥7 mm	67 (33.5)	38 (74.5)	32 (22.2)		34 (39.1)	15 (71.4)	19 (28.8)	

EMVI, extramural vascular invasion, LPLN, lateral pelvic lymph node; BMI, body mass index; CEA, carcinoembryonic antigen; CA19-9, Carbohydrate antigen19-9.

### Selection of Risk Factors for LPLN Metastasis in the Development Cohort

Based on the univariable logistic analysis, sex, grade of differentiation, gross classification, EMVI, distance of the tumor from the anal verge, cT stage, perirectal lymph node status, preoperative CA19-9 level, and largest short-axis diameter of LPLN were associated with LPLN metastasis (**Table 2**). Multivariate logistic regression analysis further identified five independent risk factors that were significantly associated with LPLN metastasis, including poor or worse differentiation (odds ratio (OR) = 2.839; 95% confidence interval (CI) = 1.185–6.804; *p* = 0.019), EMVI (OR = 3.747; 95% CI = 1.385–10.135; *p* = 0.009), distance of the tumor from the anal verge <5 cm (OR = 2.824; 95% CI = 1.074–7.431; *p* = 0.035), positive perirectal lymph nodes (OR = 4.524; 95% CI = 1.718–11.912; *p* = 0.002) and largest short-axis diameter of LPLN ≧7 mm (OR = 7.574; 95% CI = 3.216–17.837; *p* < 0.001) (**Table 2**).

**Table 2:** Univariate and multivariate analyses of risk factors for lateral pelvic lymph node metastasis in a model-development cohort.

	Univariate analysis		Multivariate analysis	
	Odds ratio (95% CI)	*p*-value	Odds ratio (95% CI)	*p*-value
Age (years)	0.854 (0.681–1.071)	0.171		
Sex				
Male	1		1	
Female	2.497 (1.316–4.736)	0.005	1.445 (0.615–3.393)	0.399
BMI (kg/m^2^)				
<25 kg/m^2^	1			
≥25 kg/m^2^	0.652 (0.325–1.309)	0.229		
Grade of differentiation				
Well/moderate	1		1	
Poor/worse	4.538 (2.312–8.908)	<0.001	**2.839 (1.185–6.804)**	**0.019**
Gross classification				
Protuberant type	1		1	
Ulcerative type	5.899 (1.353–25.729)	0.018	2.467 (0.396–15.377)	0.333
EMVI				
Negative	1		1	
Positive	4.605 (2.099–10.106)	<0.001	**3.747 (1.385–10.135)**	**0.009**
The size of tumor	0.912 (0.746–1.116)	0.372		
Distance from the anal verge				
≥5 cm	1		1	
<5 cm	3.531 (1.651–7.552)	0.001	**2.824 (1.074–7.431)**	**0.035**
cT stage				
T1–2	1		1	
T3–4	3.207 (1.187–8.667)	0.022	1.310 (0.358–4.799)	0.683
Perirectal lymph nodes status				
Negative	1		1	
Positive	7.071 (3.296–15.172)	<0.001	**4.524 (1.718–11.912)**	**0.002**
Preoperative CEA level				
<5 ng/ml	1			
≥5 ng/ml	1.730 (0.918–3.257)	0.090		
Preoperative CA19-9 level				
<37 U/ml	1		1	
≥37 U/ml	2.288 (1.143–4.580)	0.019	1.939 (0.705–5.331)	0.199
The largest short-axis diameter of LPLN				
<7 mm	1		1	
≥7 mm	8.416 (4.176–16.959)	<0.001	**7.574 (3.216–17.837)**	**<0.001**

EMVI, extramural vascular invasion; LPLN, lateral pelvic lymph node; BMI, body mass index; CEA, carcinoembryonic antigen; CA19-9, carbohydrate antigen19-9. “bolded” which means the difference was statistically signifcant.

### Construction and External Validation of the Nomogram Model

**Figure 2:**
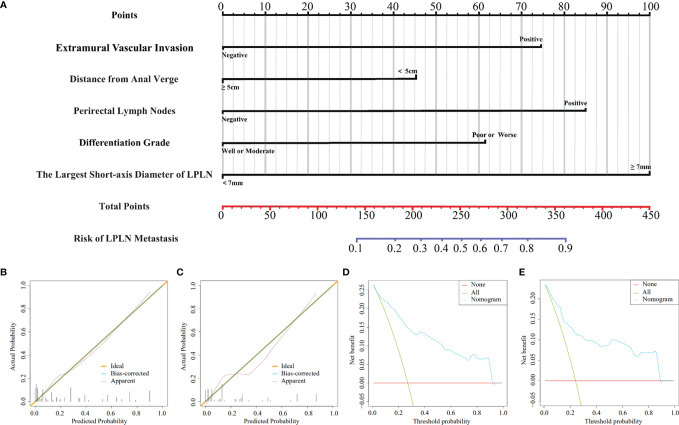
A nomogram model for predicting the risk of lateral lymph node metastasis in advanced lower rectal cancer patients **(A)**. Calibration curves for training cohort **(B)** and validation cohort **(C)**, respectively. DCA for training cohort **(D)** and validation cohort **(E)**, respectively. LPLN, lateral pelvic lymph node; DCA, decision curve analysis.

**Figure 3:**
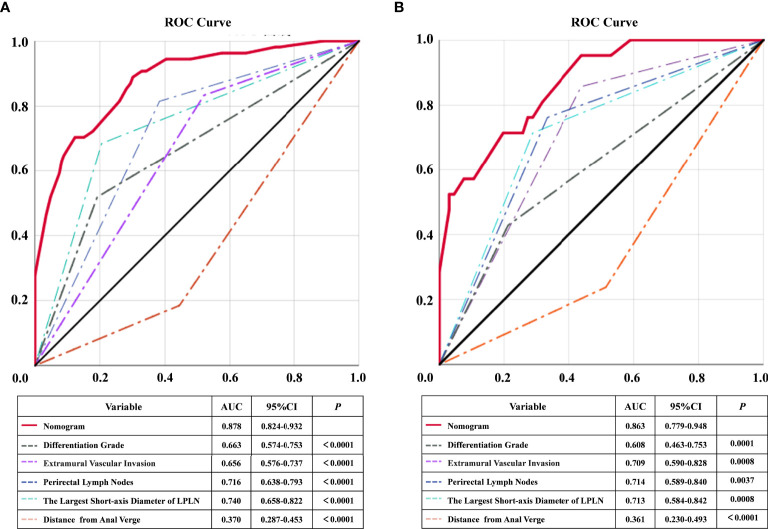
ROC and statistical comparison for each individual predictor and combined nomogram of training cohort and validation cohort. ROC, receiver operating characteristic; AUC, the area under the curve; LPLN, lateral pelvic lymph node.

Based on the above independent risk factors, a nomogram model was constructed to preoperatively predict the probability of LPLN metastasis for ALRC patients **(** **Figure 2A**). The calibration curves of the development cohort and validation cohort after external verification were generated and are illustrated in **Figures 2B, C**, respectively. The calibration curves suggested that the predicted and actual incidence rates were almost identical, which indicated that the nomogram performs well in predicting LPLN metastasis. In addition, the DCA curve showed that the nomogram model had good clinical predictive power, suggesting that the nomogram could serve as an effective diagnostic tool for predicting LPLN metastasis (**Figures 2D, E**). The ROC curve was used to further compare the discrimination performance of the comprehensive nomogram model and single risk factors in predicting the occurrence of LPLN metastasis. The results indicated that the nomogram model significantly outperformed the other factors (all *p* < 0.005), with the highest AUCs of 0.878 (95% CI, 0.824–0.932) in the training cohort and 0.863 (95% CI, 0.779–0.948) in the validation cohort (**Figures 3A, B**), which again verifies the predictive power of the nomogram model.

### Predictive Power of the Nomogram Model Scoring System

**Figure 4:**
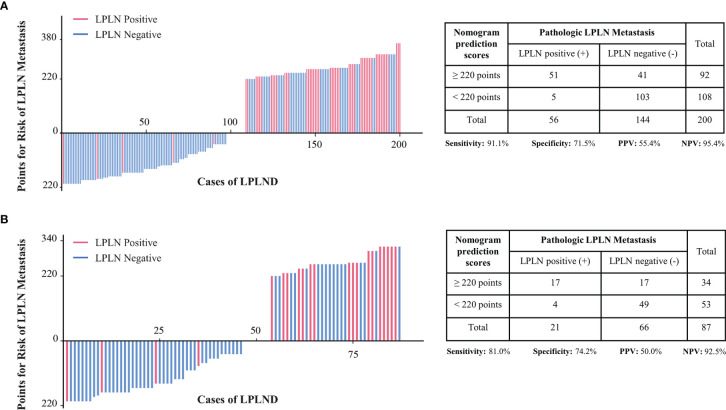
Patients in the development cohort **(A)** and the validation cohort **(B)** were divided into low-risk and high-risk groups based on the cutoff value of the total score of the nomogram. LPLN, lateral pelvic lymph node; LPLND, lateral pelvic lymph node dissection; PPV, positive predictive value; NPV, negative predictive value.

To further improve the clinical practicability of the risk quantification model, we performed risk classification based on the cutoff value (220 points) of the nomogram score, which was determined by the maximum Youden index of the nomogram ROC curve, and the patients were divided into high-risk (total points ≥220 points) and low-risk (total points <220 points) groups. In the low-risk group, the LPLN metastasis proportions in the development and external validation sets were 5/108 (4.6%) and 4/53 (7.5%), respectively, whereas the proportions were 51/92 (55.4%) and 17/34 (50.0%) in the high-risk group (**Figures 4A, B**). A higher nomogram score was often accompanied by an increased risk of LPLN metastasis. For the risk classification based on our nomogram, the sensitivity, specificity, positive predictive value (PPV), and negative predictive value (NPV) in the development set were 91.1%, 71.5%, 55.4%, and 95.4%, respectively. The sensitivity, specificity, PPV, and NPV of the validation set were 81.0%, 74.2%, 50.0%, and 92.5%, respectively.

## Discussion

Even though the addition of LPLND to TME could bring oncological benefits, the procedure is inappropriate for all patients, considering that only 7% to 23.8% of patients with ALRC have LPLN metastasis (15). Moreover, LPLND is technically demanding and has been reported to increase the incidence of some nerve-related complications, such as urinary disorders and sexual dysfunction. Thus, it is essential to identify which patients could obtain the maximum benefit-risk ratio from LPLND prior to treatment. The current study developed and externally validated a novel nomogram model, which combined preoperative MRI features and clinicopathological characteristics, to predict the risk of LPLN metastasis in patients with ALRC. The prediction model was constructed based on several independent risk factors associated with pathological LPLN metastasis, including tumor differentiation grade, tumor location, EMVI, perirectal lymph node status, and the largest short-axis diameter of LPLN, and showed excellent prediction performance in both the model development and external validation cohorts.

The radiographic features of metastatic lymph nodes have been reported by a series of studies, which included diameter criteria, such as the largest long-axis, largest short-axis, and long axis-to-short axis ratio, and morphological criteria, such as mixed-signal intensity and irregular nodal capsule border (16, 17). However, for LPLNs, the above morphological criteria were not shown to be significantly associated with pathologic LPLN metastasis by Akiyoshi et al. (18). In addition, the oncological outcomes based on the local recurrence rates reported by Kusters et al. (19) demonstrated that the size criteria were more reliable than the above morphological characteristics. Among the diameter criteria, the largest short axis of LPLN was considered the ideal parameter by multiple studies, but the optimal cutoff value for suspicious LPLN on MRI still remained controversial and varied largely between studies, ranging from 3 to 10 mm with a sensitivity of 44%–87% and a specificity of 57%–91% (20–22). In the present study, the cutoff value of the largest short-axis diameter of LPLN on MRI was determined by using the maximum Youden index of the ROC curve in the model development cohort. Based on all the above, a cutoff value of 7 mm of the largest short-axis for LPLN was used as a measure during the MRI assessment.

In addition to the above radiographic predictive factors in LPLNs, regional tumor-related imaging characteristics could be used to improve the prediction of LPLN metastasis. From previous studies, pathological mesorectal node metastasis (pN stage) has been reasonably shown to be an important risk factor for LPLN metastasis (23). Based on this, Hiyoshi et al. reported that clinical mesorectal node positivity (cN+) based on MRI could also be a predictive factor to evaluate LPLN status preoperatively (24). Moreover, a recent study by Hamabe et al. (25) reported that MRI-based EMVI positivity in rectal cancer patients was significantly associated with pathological LPLN metastasis (OR and 95% CI, 6.53 and 1.26–33.8). In addition to all the above imaging features, a number of previous studies have also found some clinicopathological characteristics significantly associated with pathological LPLN metastasis (26–28). In the present study, our results showed that poor or worse tumor differentiation and tumor distance from the anal verge <5 cm were independent risk factors associated with pathological LPLN metastasis, which was consistent with previous reports. Thus, to improve the prediction accuracy of LPLN metastasis, the above imaging features and clinicopathological factors were combined to construct the novel nomogram model.

It is well known that treatment modalities should be based on detailed pretreatment assessment and an individualized approach that considers all options to optimize the treatment of patients with rectal cancer. However, the best approach to diagnosing LPLN metastasis is unknown because the study results were inconsistent. Therefore, improvements in approaches to qualitative assessment are needed. Nakanishi et al. reported that radiomics-based prediction modeling provides an individualized risk estimation of LPLN metastasis in rectal cancer patients treated with (chemo)radiotherapy (29). Amano et al. reported that the combination of CT, MRI, and PET/CT did not show better predictive value for LPLN metastasis than a single imaging assessment alone (30). Although these diagnostic efficacies are relatively high, the influence of clinicopathological factors is not considered. These complete radiographic evaluations are not convenient for clinical application and have not yet been validated in further external sets. A large Japanese multicentric study constructed a model for predicting LPLN metastasis based on clinicopathological factors and radiographic features (31). However, the C-index of the model was 0.74, which indicates moderate accuracy in predicting LPLN metastasis. In our study, the nomogram showed good prediction efficiency in both the model development set (AUC = 0.88) and the external validation set (AUC = 0.86). Moreover, to further improve the clinical practicability of the nomogram prediction system, we calculated the optimal cut-off value of the prediction model (220 points). According to this cut-off value, the NPVs of the diagnostic prediction model were 95.4% and 92.5% in the model development and validation cohorts, respectively. The NPV is high, and it is considered an effective evaluation method to determine the need to perform LPLND, which means that patients who were diagnosed as low risk could avoid undergoing extensive surgery, which could impact the postoperative quality of life. On the other hand, for high-risk patients, the clinician should consider not only the probability of LPLN metastasis but also surgical comorbidity, postoperative quality of life, and the patient’s opinion. With as much information as possible, the patient can be further supported to become more actively involved in decision-making, which may improve the patient’s adherence to treatment. Thus, this nomogram may be used to help colorectal surgeons make clinical decisions for ALRC patients.

This study has some limitations. First, the sample size of the model development set, including 200 patients, was not large enough. A more extensive and prospective dataset is needed to generalize the performance of the LPLN metastasis prediction model. Second, nCRT caused a decreased number of LPLNs detected and pathological transformation of LPLNs, which may lead to a certain bias. However, the sample of patients who underwent nCRT+TME+LPLND was too small to allow further subgroup analysis. Therefore, the change in LPLNs before and after nCRT was not further assessed. Moreover, if the changes in LPLNs after nCRT were used as indicators, the predictive power of the model, especially its specificity, may be further improved.

Despite these drawbacks, we believe our study findings are valuable. In our study, LPLN metastasis was predicted preoperatively through a combination of imaging assessment and clinicopathological characteristics, which provides a new perspective for the comprehensive diagnosis and treatment of LPLN metastasis. Patients should be classified depending on their risk of developing LPLN metastasis to select the best option to manage the pelvic compartment. In patients who have a low risk of LPLN metastasis, undergoing nCRT+TME may be sufficient to avoid overtreatment. Patients who have a high risk of LPLN metastasis may need to undergo nCRT + TME + LPLND to achieve better local control.

In conclusion, we present a novel, externally validated model to predict the risk of LPLN metastasis, which could provide an individual prediction of LPLN metastasis with good accuracy and serve as a useful guide in patient management.

## Data Availability Statement

The raw data supporting the conclusions of this article will be made available by the authors, without undue reservation.

## Ethics Statement

This study was reviewed and approved by The First Affiliated Hospital of Xi’an Jiaotong University Ethics Committee, 2019-ZD-04 and Peking University First Hospital Biomedical Sciences Ethics Committee, 2021-351. Written informed consent was obtained from the individual(s) for the publication of any potentially identifiable images or data included in this article.

## Author Contributions

LZ and FS: data collection, formal analysis, investigation, methodology, project administration, software, validation, and writing original. CH, ZZ, JL, and RL: data curation, formal analysis, investigation, methodology, review, and editing. JS and JT: project administration, validation, review, editing, and supervision. All authors contributed to the article and approved the submitted version.

## Funding

The study was supported by the National Natural Science Foundation of China (81870380, 81702362).

## Conflict of Interest

The authors declare that the research was conducted in the absence of any commercial or financial relationships that could be construed as a potential conflict of interest.

## Publisher’s Note

All claims expressed in this article are solely those of the authors and do not necessarily represent those of their affiliated organizations, or those of the publisher, the editors and the reviewers. Any product that may be evaluated in this article, or claim that may be made by its manufacturer, is not guaranteed or endorsed by the publisher.
