# Supplementary material for: Development and External Validation of a Preoperative Nomogram for Predicting Lateral Pelvic Lymph Node Metastasis in Patients With Advanced Lower Rectal Cancer
Source: Front Oncol. 2022 Jul 8;12:930942. doi: 10.3389/fonc.2022.930942 (PMC9307891; doi:10.3389/fonc.2022.930942)
Supplement: Supplementary file 1 [file DataSheet_1.docx]

**Development and external validation of A** **Preoperative Nomogram for Predicting the Lateral Pelvic Lymph Node Metastasis in Patients with Advanced Lower Rectal Cancer**

Zhang et al.

(Supplementary materials)

**Receiver operating characteristic (ROC) curve analysis for evaluation of cut-off values**

The optimal cut-off values of continuity variables were determined by the ROC curve.

**
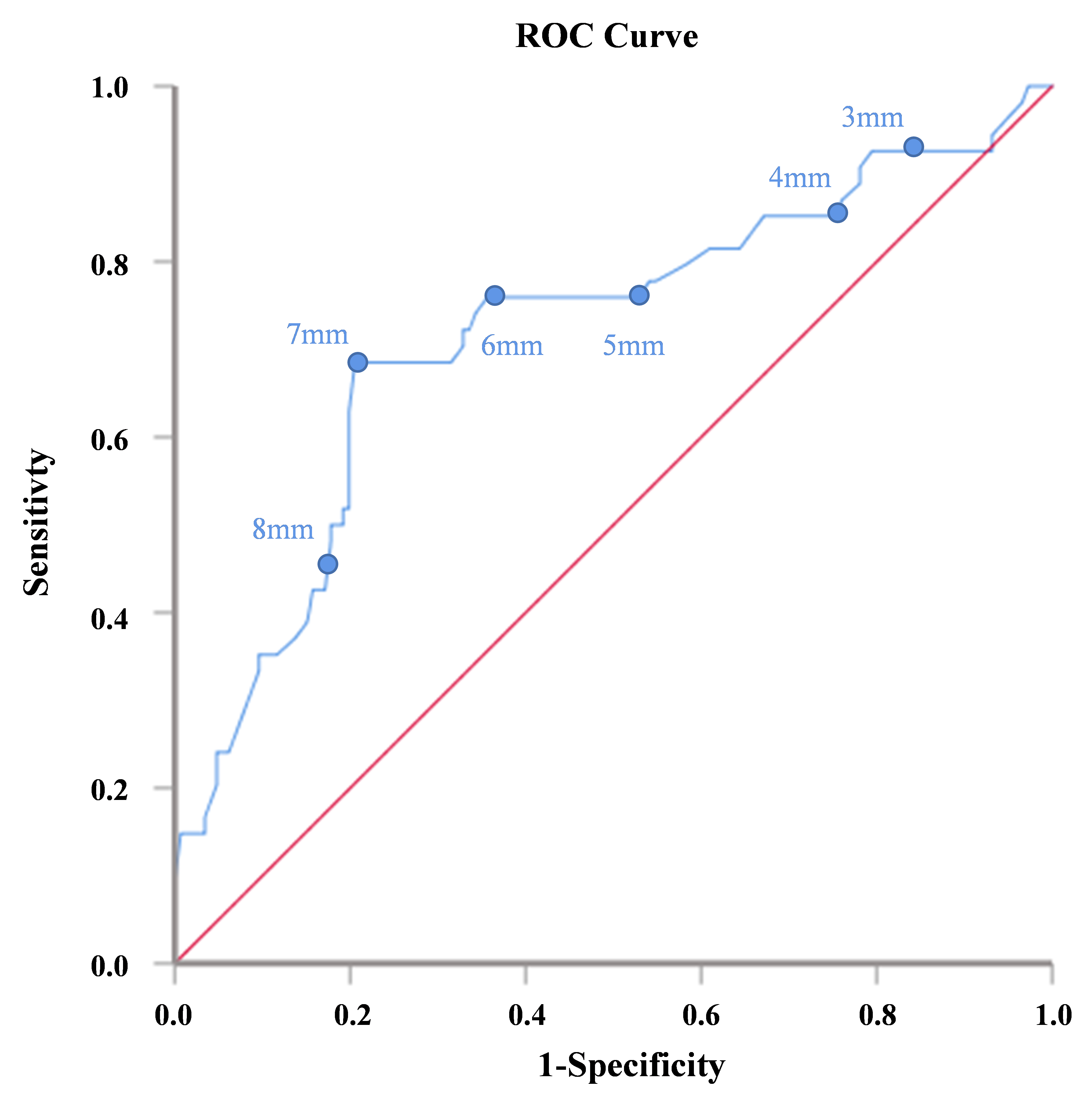
**

**Figure S1.** The receiver operating characteristic curve for the per-patient size of the largest short-axis diameter of lateral lymph nodes to diagnose lateral lymph nodes metastasis

The ROC curve for the per-patient size of the largest short-axis diameter of lateral lymph nodes were shown in **Figue S1**. The area under the curve (AUC) was 0.718, 95%CI (0.632-0.804), indicating moderate accuracy on per-patient analysis. The optimal value of the largest short-axis diameter was 7 mm. Sensitivity, specificity, positive predictive values, and negative predictive values, and accuracy of per-patient evaluation according to this cut-off value were 68.5 %, 77.4 %, 52.9 %, 87.0 % and 75%, respectively.

**
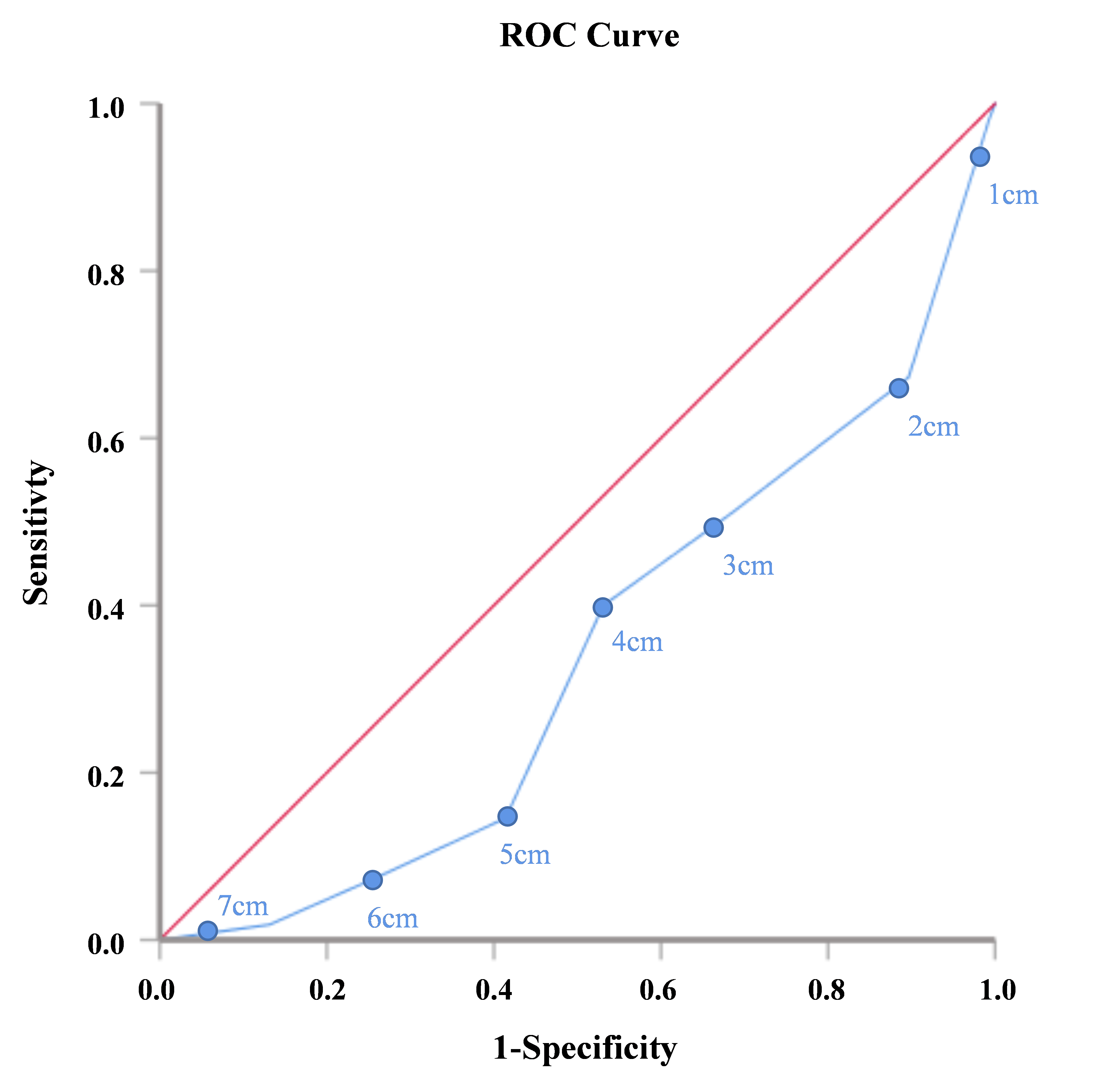
**

**Figure S2.** The receiver operating characteristic curve for the per-patient of the distance of the tumor from the anal verge to diagnose lateral lymph nodes metastasis

The ROC curve for the per-patient of the distance of the tumor from the anal verge were shown in **Figue S2**. The AUC was 0.383, 95%CI (0.298-0.468), indicating low accuracy on per-patient analysis. The optimal cutoff value of the distance of the tumor from the anal verge was 5 cm. Sensitivity, specificity, positive predictive values, and negative predictive values, and accuracy of per-patient evaluation according to this cut-off value were 81.5 %, 41.1 %, 33.8 % , 85.7 % and 52% , respectively.
